# On the Action of Medicines

**Published:** 1848-06

**Authors:** 


*On The Action of Medicines*, by JAMES BLAKE, M. D., Professor of Anatomy in the St. Louis University.

* In the last two memoirs on the action of medicine, which I published in this journal, I have endeavored to show that the strongest arguments that have been brought forward in the support of supposed sympathetic action of substances, taken into the stomach, or applied to any part of the economy, have been adduced on insufficient data, and that when properly and carefully observed, these very facts that are supposed to support the theory of the sympathetic action of medicines, furnish us with the most striking proofs that it is necessary that they should enter into the blood, before they can produce their effects, and this being the case with that class of agents which has been always held up as the most signally sympathetic in its action, it is but reasonable to suppose that many, if not most of our remedial agents, modify the animal economy by entering into the blood, and that therefore, in the scientific investigation of the action of medicines, a point of the greatest interest is, to endeavor to ascertain the effects produced on the blood, by the substances that are thus mixed with it, and also the changes produced on the tissues of the body by their contact with the substances that are thus brought to them. I would not, however be understood as advocating any special doctrine as regards the action of medicines, or asserting that some do not produce their effects on the animal by a local action they exert on the part to which they are applied, for my experiments, as far as they have been carried, do not at all justify any such deduction, but they certainly tend to point out that the absorption of substances into the blood is a phenomenon that takes with much greater facility, and much more frequently than had been previously imagined. In the present days of reviving humoralism, this fact will probably serve to give an additional impulse to the spirit of exaggeration, that would lead the adopters of these revived notions, to attribute all the morbid phenomena of the animal economy, to changes taking place in the fluids; but whilst at the same time that I believe that a great many of our medicines act when taken into the blood, and through changes produced in that fluid, yet I think the exclusive doctrine of the humoral action of medicines, would be as ridiculous as that which supposes, that when a man sneezes after taking snuff, or has an attack of epilepsy from irritation in any other part of the mucous membrane, that the sneezing and the epilepsy are the result of changes produced in the blood. But to return to the subject of the action of medicine: It is evident from the facts already brought forward in my previous memoirs, that the chief object in my scientific investigation of this point, must lead to an enquiry into the changes produced by the substances in the blood and tissues of the animal economy, with which they are brought into contact. Simple as this problem might at first sight appear, yet, on a closer investigation, it will be found surrounded with difficulties. Could we be contented with mere Empirical knowledge furnished by the observation of the effects produced by our more usual medicinal agents on diseased subjects, it would be an easy task to lay before the profession a vast amount of facts, which however would be but little calculated to command their attention, as they would necessarily be encumbered with all the fallacies inherent to every investigation that is not controled by the true spirit of scientific research. Unfortunately in the present state of our art,

*See Buffalo Medical Journal, Vol. 3.

the direct application of the results furnished by every scientific course of enquiry on this subject, is not to be looked for. The only path by which we can hope to extend our knowledge as a science is, by gradually proceeding, step by step, from the known to the unknown ; and, although much has been done of late years towards diminishing the immense distance which formerly separated our certain scientific arguments in physiology and pathology, from the complicated phenomena of disease, yet it must not be expected that discoveries in the more purely scientific branches of the science of Biology, can as yet cast but a faint gleam across the wide space which still intervenes between the confines of Physiology as a science, and the point where the data it furnishes can be brought to bear on that most complicated of all its problems—the human organism in a state of disease. The attention that has been given of late years to the purely chemical and physical phenomena of living beings—the extended views that have led the scientific Physiologist to carry his researches through the whole order of living beings, and to attempt to seize the phenomena which are peculiar to them, under the simplest forms in which they present themselves — the light has been thrown by chemistry on many of the reactions which are taking place in the living organisms, have fortunately done much towards removing those barriers which would exclude these reactions from within the pale of our experimental researches. Not only has the day gone by when the scalpel of the anatomist was forbidden to sound the hallowed textures of the human frame, but the moment is at last arrived when the chemist may enter the more sacred sanctuary of the living organism, and with the aid of his reagents, attempt to elucidate the interesting phenomena of which it is the seat. It would be a useless task to attempt to prove that these phenomena are the result of reactions taking place between the elements of which the organism is composed, and which reactions must be themselves due to the composition and properties of the different elements of which it is made up. I think that the belief that such is the case, is very widely diffused amongst the thinking portion of the profession, and in the present day, would scarcely be denied by any physiologist. Admitting, therefore, that such is the case, the primary question which presents itself, in an inquiry into the means of modifying these properties is, what are the reactions which the elements of the living animal present, when brought into contact or submitted to the influence of our various reagents? Simple and self-evident as the question appears, it is astonishing that its solution should, under a scientific point of view, have been attempted by so small a number of those who have occupied themselves in the study of the influence of various agents on the animal economy; for although the question of determining the properties of the elements of a living being is certainly more complicated than that of determining the properties of an inorganic compound, this consideration can afford no reason why we should sit down in despair before the problem as insoluble, and not even apply to its elucidation those means which would immediately present themselves to us if our researches were directed on phenomena less complicated. Had Davy been led to despair of even decomposing the Earths, on account of the difficulty of his task, where would now be the brilliant discoveries which have thrown so much light on chemistry during the last thirty years. The more difficult the task, the more powerful and varied the means he brought to bear on it. The problems which present themselves to the physiologist are, undoubtedly, far more complicated than those which fell under the observation of the chemist; but this greater complication should only afford stronger incentives for us to apply to their solution all those means which the great advance of the collateral sciences have placed at our command. Of these, the most important are undoubtedly to be derived from an investigation of the reactions which are produced in the living organism, by the various chemical reagents with which it can be brought into contact.

Looking on the living organism, as a whole, we find it, in the higher classes of animals, presenting us with various phenomena, which are, undoubtedly depending on changes constantly taking place in the composition of its elements, or with the forms which they assume.

It is not my intention, in the present paper, to touch on the latter of these subjects. Morphology is already attracting, perhaps, too large a share of attention from physiologists, particularly when it is remembered that it is principally, if not entirely, to the morphological appearances that are present by structures that no longer form a part of the living organism, that their scientific relations are confined, and should subsequent research prove, that the morphological relations of the elements of the blood and tissues, are submitted to as important variations after death, as are the chemical properties of these substances, it will then be found that the great amount of labor that has been expended on this branch of Physiology, must necessarily be less fertile in scientific results, than its almost exclusive cultivation would lead us to expect. As regards the chemical phenomena of which the living organism is the seat, I believe that they present us with a wide and fertile field of research, and one in which the discoveries that are made are likely to throw considerable light on some of the more interesting phenomena of living beings. A clear consideration of the nature of the problem to be solved, at once indicates the means that are to be applied to its solution—we want to discover what are the properties of the fluids and the solids of the animal economy, whilst still forming part of the living organism. Had we the same solids and fluids to deal with, out of the body, we should evidently attempt to discover their chemical properties, by submitting them to various chemical reagents, in the same manner as we should proceed, if we had to investigate an inorganic compound. Unfortunately, however, the animal fluids and solids, when removed from the living body, are no longer the same, or possessed of the same properties, as when forming part of the living fabric, and it is only, therefore, whilst forming part of the living being, that we can hope to be able to ascertain the properties of the elements of which it is composed. What course, therefore, has the physiologist to pursue, in order to arrive at a knowledge of facts that are of such vast importance to him, but to endeavor to bring these different reagents into the veins and arteries of a living animal? This affords a ready means of ascertaining, in what manner the elements of which it is composed, are modified by their contact with these reagents; and, although the visible effects produced in this, as in many other chemical reactions, not directly apparent to our senses, yet we possess in the various functional lesions that are produced, valuable indications of changes, that must, by their delicacy, have otherwise escaped our observation.

As it is from the muscular contractions produced in the legs of a frog by a slight current of electricity, that we possess the most delicate test for the passage of an electric current, so I believe it will be found, that in the phenomena produced by our various chemical reagents, on living beings, we shall have the most delicate test of the various chemical changes that these reagents produce in the different parts of which they are composed. An example, however, will perhaps better explain my meaning: if it is found, for example, that a single grain of chloride of platinum introduced directly into the blood of an animal, is capable of suddenly arresting the action of the heart, it is evident that this small quantity of our reagent has produced so great a change, either in the properties of the blood, or in those of the tissues of the heart, that this organ is incapable of continuing its functions: and to give another illustration from the same experiment: should it on further inquiry be found that, it is on the blood that this substance acts, we obtain a valuable indication in what direction we are to look for a solution of the important relations that connect the phenomena of muscular irritability, with the composition and properties of the blood. It is probable that no more delicate test of the changes produced by so small a quantity of an apparently inert chemical substance in the whole mass of the blood, could be discovered, than this marked functional disturbance produced in the action of the heart.

Whilst therefore on the one hand, the determination of the changes which are produced in the living organism by our various reagents, are in manner concealed from our view, yet whilst these changes are not carried so far as to destroy life, their existence is indicated by the phenomena they produce on the living organism, which itself affords a most delicate means of ascertaining their existence.

These considerations will, I trust, be an excuse for my bringing forward in the following pages, a number of facts which have been obtained by direct experimental enquiry of the effects produced by various reagents when introduced directly into the blood of a living animal. Although these facts might not appear to have a direct bearing on the "Action of Medicines," yet, under a general point of view, our medicines are nothing more or less, than means by which we produce certain modifications in the animal economy, and in considering an animal as composed of different elements, on the properties of which elements must be dependent the phenomena, both vital and physical, of which it is the seat, it is evident that it is only by submitting these elements, whilst still forming part of the living animal, to the action of our various reagents, that we can hope to arrive at a knowledge of their properties, and by the laws that govern their reactions. But to return from these general observations, which have already, perhaps, been protracted to too great a length, and which I should not have thought it necesary so much to insist on, were it not that I believe that this branch of experimental philosophy has not atracted that share of attention which it deserves, on account of the true spirit in which it should be pursued not being generally understood, and also that the advantages which its pursuit promises to afford, are not duly appreciated.

There are two points of view under which the experiments I have to bring forward might be related. The first, as regards the chemical relations of the substances employed; the second, as regards their therapeutical properties. As, however, many of the substances experimented with are not such as are commonly used in medicine, I shall notice the action of the substances principally as regards their chemical relation to each other.

It must not be forgotten that these experiments were performed by introducing the substances experimented with directly into the blood, and, therefore, the results they furnish are, in many instances, very different from those which follow the introduction of the same substances into the stomach, and will not, therefore, have in most instances any direct practical bearing on medicine, but I shall, however, endeavor to compare, as far as possible, the effects that have followed this method of administering these substances, with those that are found to be produced by them, when given in the ordinary way; and although some these effects are often directly contrary to what, from our knowledge of the ordinary action of these substances, we might have anticipated, yet, these discrepancies render, I consider, the subject the more interesting, as pointing out a field in which future researches cannot fail to throw much light.

The manner in which the experiments have been performed, is by injecting solutions of the substances used, either into the jugular vein, or into the axillary artery. When this latter method was employed, the tube connected with the syringe was introduced into the artery, with its joint towards the heart, so as to force the fluid into the aorta, and by this means to cause it to be diffused generally through the system, and thus ascertain its effects on the circulation without being brought directly into contact with the heart. The order I propose to follow in relating these experiments, is to consider together the effects produced by the different compounds of the same substance. The advantages of this arrangement will be apparent as we proceed, and will bring together substances, the physiological action of which is analogous. I shall first relate the experiments that have been performed with potass and the salts of this base. The injection into the jugular vein of a dog, of three grains pure potass, dissolved in six drachms of water, was followed by expressions of pain and slight increase in the rapidity of the heart's action, about fourteen seconds after the injection, or at the moment when the blood containing the solution would be circulating over it: the respiratory movements were quicker, and rather laboured; there were also slight tremblings of the muscles, and what might be considered as a spasm. The pressure of the blood in the arterial system was increased, but not to any great extent. The subsequent injection of six grains of the alkali, gave rise to complete paralysis of the heart.

The use of the hemadynamometer, which was connected with the arterial system, permitted me to ascertain the state of the circulation with the greatest accuracy, and it plainly indicated that there was no pulsation of the heart subsequent to twelve seconds after the injection of the salt. The respiratory movements continued pretty regularly, for two minutes after the heart's action had ceased; they then became much slower, but a distinct expansion of the thorax took place three minutes and a quarter after the action of the heart had been arrested. On opening the thorax immediately on the cessation of respiratory movements, the heart was found perfectly still, nor would it contract on the application of stimuli. There appeared to be, however, a slight movement on that part of the surface corresponding to the interventricular septum; this, however, soon ceased. Both cavities were distended with blood—that in the right side was dark and coagulated freely—that in the left side was scarlet, and was coagulated very firmly, more so than I have ever noticed it, for on removing the coagulum from the heart, the clot that filled the aorta, as far as its division into the iliacs, came out with it, although the animal had been dead not than three minutes. more

In another experiment that was made, to ascertain the effects of this substance on the general circulation, independently of its direct influence on the heart, a solution containing four grains, dissolved in about six drachms of water, was injected into the aorta through the axillary artery. In four seconds the pressure in the arterial system became enormously increased, the column of mercury in the hemadynamometer rising as high as fourteen inches. This great increase of pressure in the arteries, must be owing to the presence of the alkali in the blood, rendering its passage through the capillaries, more difficult, and as the heart continued pumping blood into the arterial system, and its exit was at the same time impeded, the acumulation of blood in the arteries was sufficient to produce this great pressure on their parieties. After a few seconds, the increased pressure appeared sufficient to overcome the obstruction, and the blood containing the salt passed on. Ten grains introduced into the aorta, led to the same obstruction to the passage of the blood through the systemic capillaries, but the increased pressure in the arterial system soon overcame it, and the blood containing the alkali was carried on, until it reached the heart, and became circulated over its parieties. This was immediately followed by the cessation of the heart's action, which took place about thirty seconds after the injection had been introduced into the aorta. As regards the general symptoms that follow the introduction of a solution of potass into the veins, they would seem to indicate an action on the nervous system; but, owing to the substance acting so fatally on the irritability of the heart, its effects on the nervous system are not very strongly marked, death always taking place by the local action of the substance on the heart. When injected in small quantities into the blood, there is generally an an expression of pain immediately on injection being pushed, and before the substance can have had time to penetrate farther than the right cavities of the heart, for this expression of pain takes place about three or four seconds after the injection of the substance into the veins. Its action on the nervous system is shown by its giving rise to spasmodic contraction of the muscles, which, however, are never carried so far as to interfere to any extent with the respiratory movements. There is nothing like narcotism or loss of sensibility, and in every case in which I have experimented with this substance, death has been caused by its action on the heart.

If we now attempt to analyze the facts furnished by the above experiments, and compare them to the toxicological and therapeutical effects of potass, when taken into the stomach, we shall find one or two interesting facts calling for remark. In the first place, as regards the toxicological effects of this substance, it is extremely probable from its causticity, and from the manner in which it destroys the tissues with which it is brought into contact, that it is rarely absorbed with the blood, at least when taken in the form of a concentrated solution. In all cases of poisoning by this substance, that are recorded, it would appear that death was the result of its local action—and the experiments of Orfila and Bretonneau, who administered it to dogs in large doses, would seem to show that, however large the dose, it never gave rise to symptoms which would lead us to expect that it is absorbed in any quantity into the blood. There can be no doubt but that its action on the tissues of the stomach is such as to disorganize them to such an extent that circulation is immediately arrested in the part to which it is applied, and thus there remains no channel by which it can find its way into the system. It is not, therefore, surprising that in cases of poisoning with this alkali, we meet with no symptoms that would indicate its presence in the blood. We shall see presently that this is not the case with the carbonates of this base, which not exerting so destructive an action on the animal tissues, are absorbed and show signs of their presence in the blood, by the particular action they exert on the heart. When given in a diluted form, the alkali is best known for its use in certain cases of indigestion, in which acidity of the stomach is a marked symptom. In this case, it probably acts topically, not merely as neutralizing the excess of acid, but also directly on the mucous membrane itself, as a local stimulant, producing effects analogous to those which follow the application of diluted caustics to the mucous membrane of the eye and of the urethra: but there are undoubtedly cases in which it is absorbed into the blood, and in which it produces its therapeutical action in this manner. I allude to those instances in which it modifies the urinary secretion. In these cases, however, its accumulation to a deleterious extent in the blood, is guarded against by its action as a diuretic, so that it is undoubtedly eliminated by the medicines from the blood almost as rapidly as it is absorbed. The alkalies, particularly potash, have been recommended in those cases of inflammation which have a tendency to give rise to the formation of membranous exudations, such as croup. I have no experience as to their effects in these diseases, but the facts brought forward, in the above experiments, show that the theoretical views on which the above practice has been recommended, must be false, for these views are founded on the supposition that these alkalies have a tendency to diminish the plasticity of the blood, and thus counteract the tendency which this fluid has in these cases to pour out a plastic exudation. It is proved, however, that the presence of potash in the blood has an influence directly the opposite of that which has been attributed to it, and we must conclude as far as increased plasticity of the blood is concerned as an element in these diseases, this remedy must be absolutely prejudicial. The physiological effects of the alkali when injected into the veins, present us with one or two striking phenomena. The expression of pain immediately on its introduction into the veins, would seem to show that the parieties of the veins, are supplied with nerves capable of conveying sensations, or, at least, that some such nerves must exist in these vessels, or in the heart. This fact is opposed to the generally received opinion on this point; but, from this as well as from other experiments, I am inclined to believe that the parieties of the blood vessels are supplied with nerves capable of transmitting sensations excited by stimuli. The facility with which the blood containing the alkali passes through the pulmonary capillaries, compared with the obstruction it meets with in the systemic capillaries, is an interesting fact: it furnishes us with a marked example of the influence which the character of the blood may exert in its passage through these vessels, but even does more than this, for it shows that blood possessing certain properties can pass through the capillaries of one organ without producing any obstruction, whilst its passage through the capillaries of another organ, may be almost arrested, or at least greatly impeded. This fact is one which may tend to throw light on the partial changes which pathology teaches are constantly taking place in the quantity of blood circulating through different organs, and may also elucidate the object of secretion. The action of potass on the heart, gives us a striking proof of the rapidity of the circulation; but to this I have already alluded in a previous article. But we have seen an interesting proof the intimate connection that exists between the composition of the blood and the functions of the organs supplied by it. The presence of so small a quantity as six grains of potass in the blood was sufficient suddenly to destroy the irritability of the heart, and thus to produce death. The opinion of Orfila, as to the cause of death in these cases being due to the coagulation of the blood, is one which is disproved.—*St. Louis Med. Jour.*

